# Supplementary material for: Single-Cell RNA-Seq Reveals Conserved Cellular Communication Mechanisms Governing Ocular Lineage Specification from Human iPS Cells
Source: Cells. 2026 Jan 7;15(2):104. doi: 10.3390/cells15020104 (PMC12839081; doi:10.3390/cells15020104)
Supplement: Supplementary file 1 [file cells-15-00104-s001.zip › REVISED_cells-3961581-supplementary.pdf]

Supplementary information for:

Single-cell RNA-Seq Reveals Conserved Cellular Communication Mechanisms Governing Ocular Lineage Specification from Human iPS Cells

Laura Howard[1,2], Yuki Ishikawa[3,4], Rei Kamuro[4,5], Tomohiko Katayama[3,4], Kiranjit K. Bains[1], Matthew J. Hill[2], Derek J. Blake[2], Sung-Joon Park[6], Ryuhei Hayashi[3,4], Andrew J. Quantock[1*], Kohji Nishida[4,5*]

[1]School of Optometry and Vision Sciences, Cardiff University, Cardiff, Wales, UK
[2]Centre for Neuropsychiatric Genetics and Genomics, School of Medicine, Cardiff University, Cardiff, Wales, UK
[3]Department of Stem Cells and Applied Medicine, Osaka University Graduate School of Medicine, Osaka, Japan
[4]Department of Ophthalmology, Osaka University Graduate School of Medicine, Osaka, Japan
[5]Institute for Open and Transdisciplinary Research Initiatives, Osaka University, Osaka, Japan
[6]Laboratory of AI Genome Informatics, Department of Frontier Research and Development, Kazusa DNA Research Institute, Chiba, Japan

* Correspondence: QuantockAJ@cardiff.ac.uk; knishida@ophthal.med.osaka-u.ac.jp

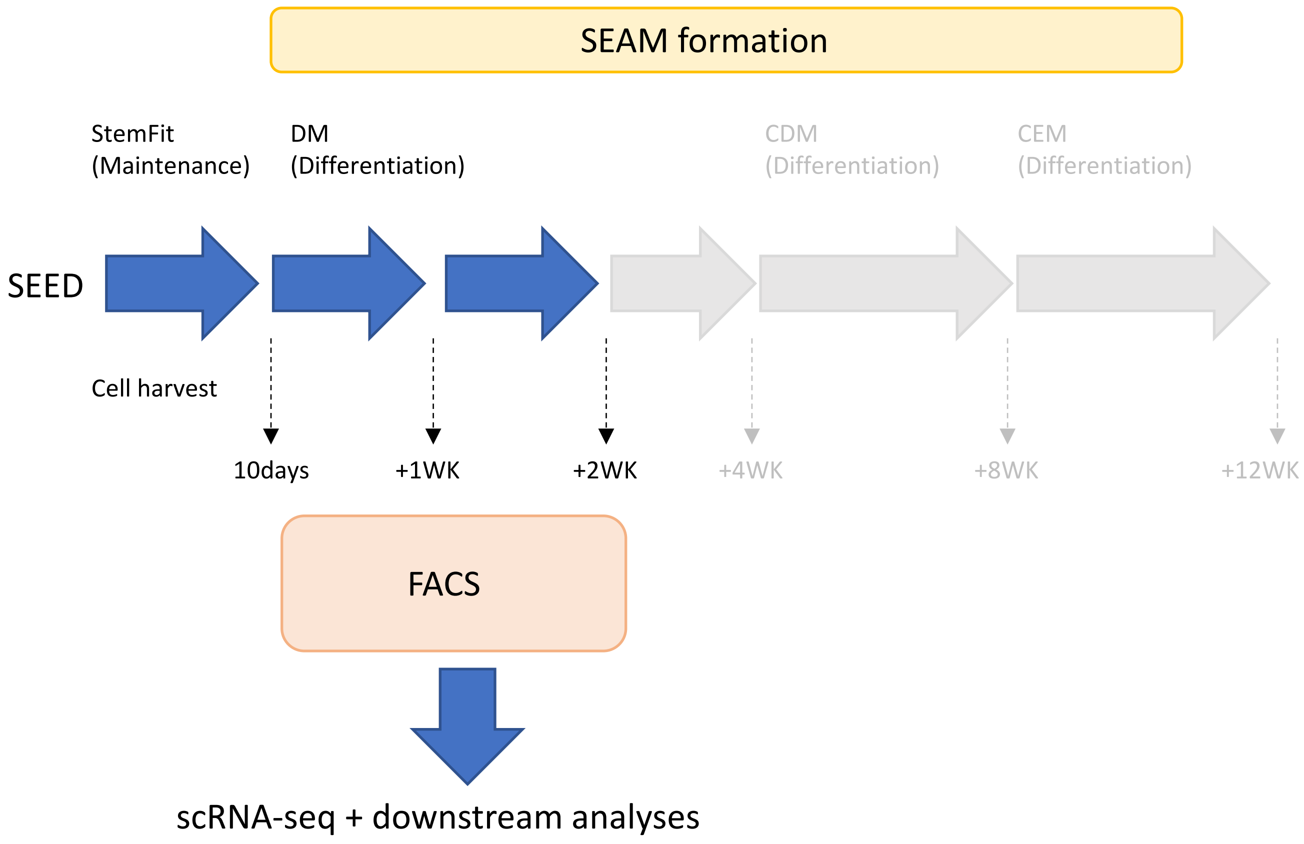

Figure S1: Experimental strategy to generate and sort hiPSC-derived SEAMs for scRNA analyses. hiPSCs were cultured on LN511E8-coated dishes in StemFit maintenance medium for 10 days, after which the medium was changed to differentiation medium to promote SEAM formation. For this study, we analysed data generated from cells at the +10days, +1WK and +2WK timepoints only, originally collected as part of a larger study conducted over 12 weeks (shown in grey).

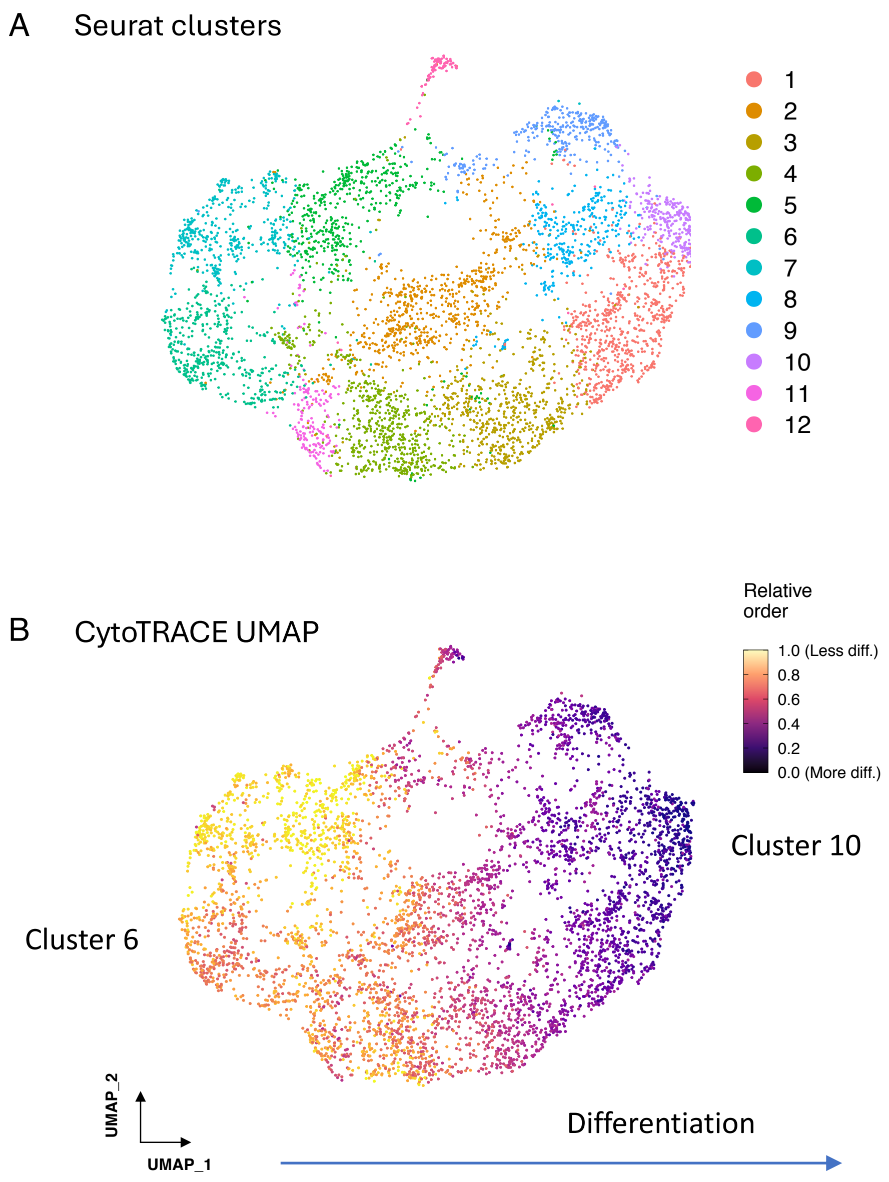

Figure S2: Seurat clustering and developmental potency scores in +10day hiPSC/SEAMs. (A) UMAP representation of +10day hiPSC/SEAMs. (B) CytoTRACE2 analysis showing relative cellular differentiation status.

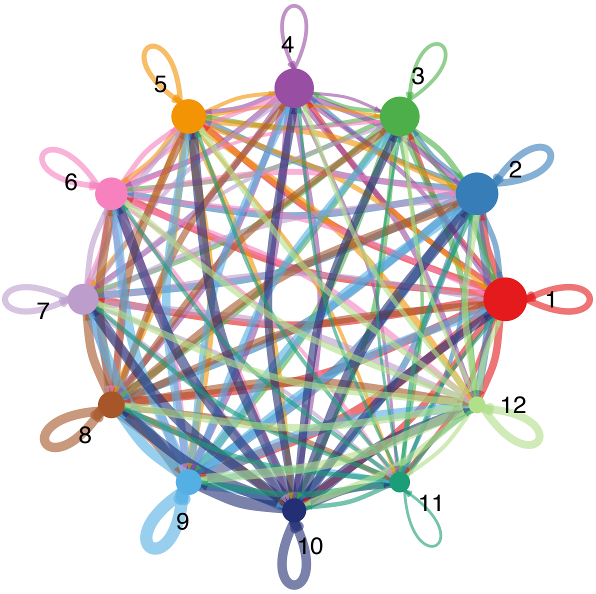

Figure S3: Aggregated cell-cell communication network in 10-day-old hiPSC/SEAMs. Cell-cell communication network between Seurat clusters pre-differentiation. The interactions to/from each cluster node are illustrated by netVisual_circle, with parameter CellChat@net$count.

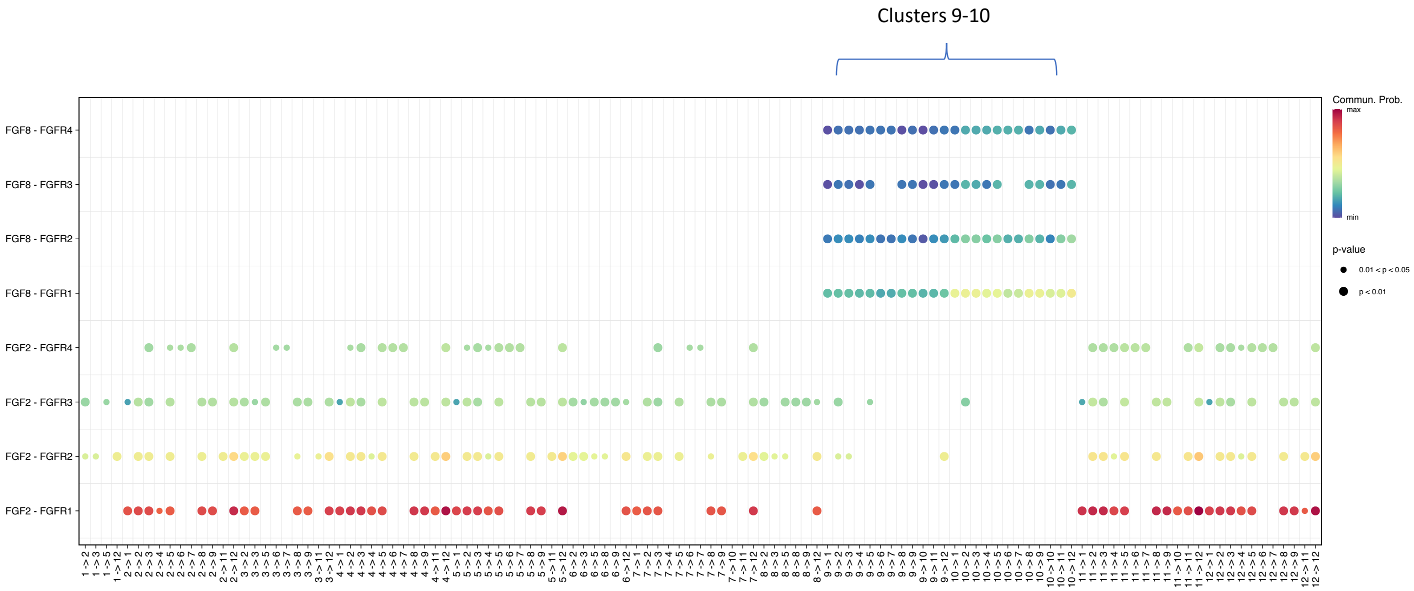

Figure S4: CellChat inference of FGF signalling in 10-day-old hiPSC/SEAMs. Bubble plot showing significant FGF pathway interactions (L-R pairs) in 10-day-old hiPSC/SEAMs. Clusters 9-10 represent the more differentiated cells in the dataset.

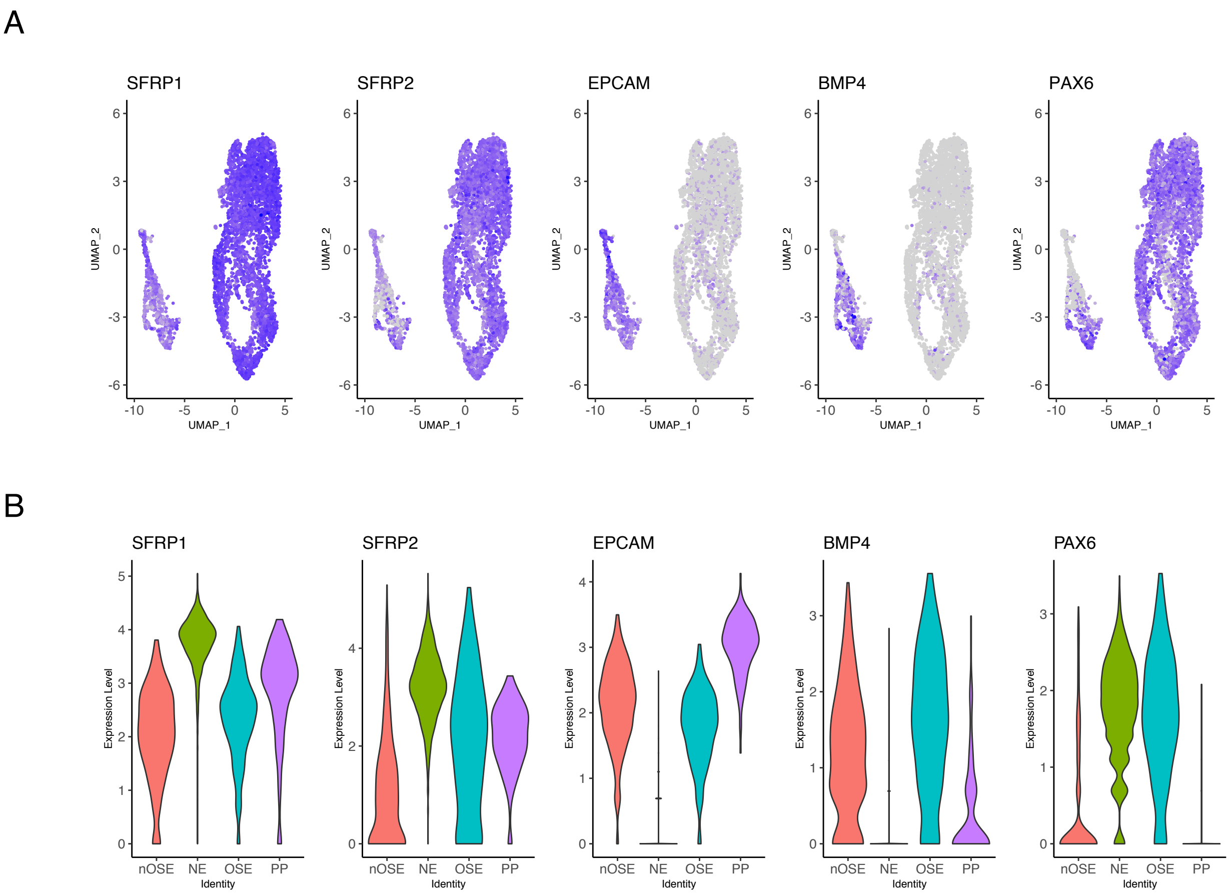

Figure S5: *SFRP1* and *SFRP2* in +1WK SEAMS. (A) Feature Plots showing expression of *SFRP1* and *SFRP2* expression in +1WK SEAMs. *EPCAM* and *BMP4* mark prospective non-neural ectoderm. High *PAX6* expression denotes ocular cells. (B) Violin plots showing expression levels. OSE, ocular surface ectoderm; nOSE, non-ocular surface ectoderm; NE, neuroectoderm; PP, pluripotent cells.

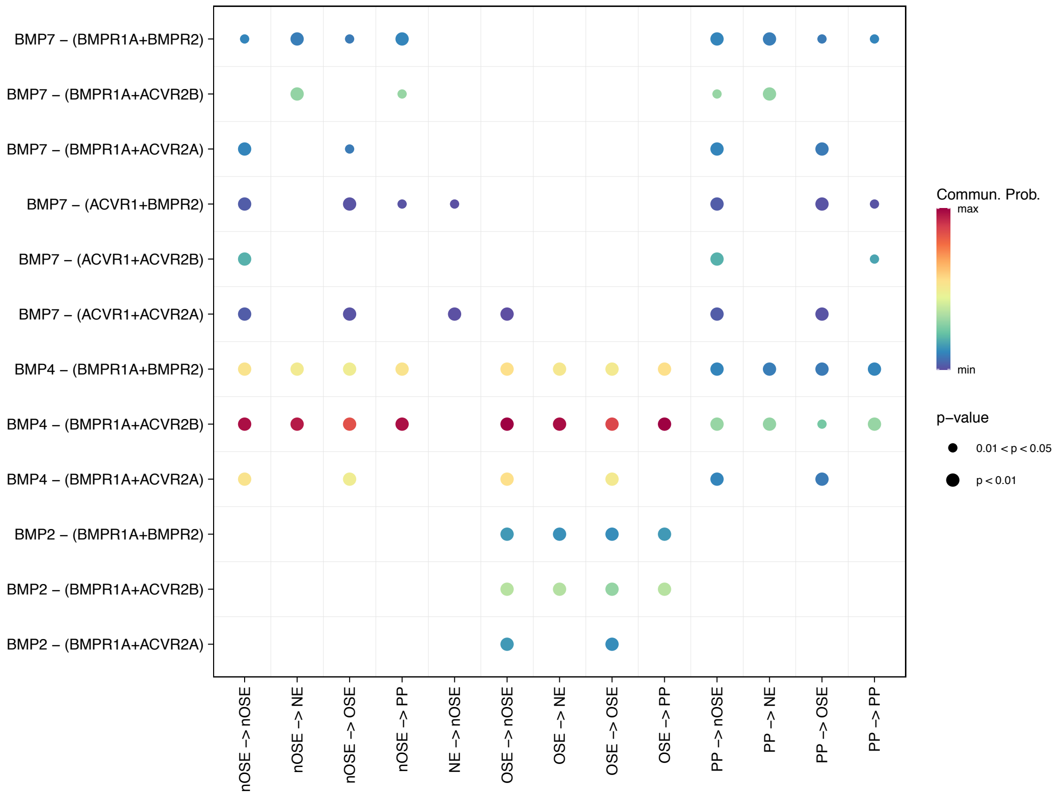

Figure S6: BMP receptor-ligand communication probabilities in +1WK SEAMs. Bubble plot showing communication probabilities between specific BMP receptor-ligand pairs. OSE, ocular surface ectoderm; nOSE, non-ocular surface ectoderm; NE, neuroectoderm; PP, pluripotent cells.

A

B

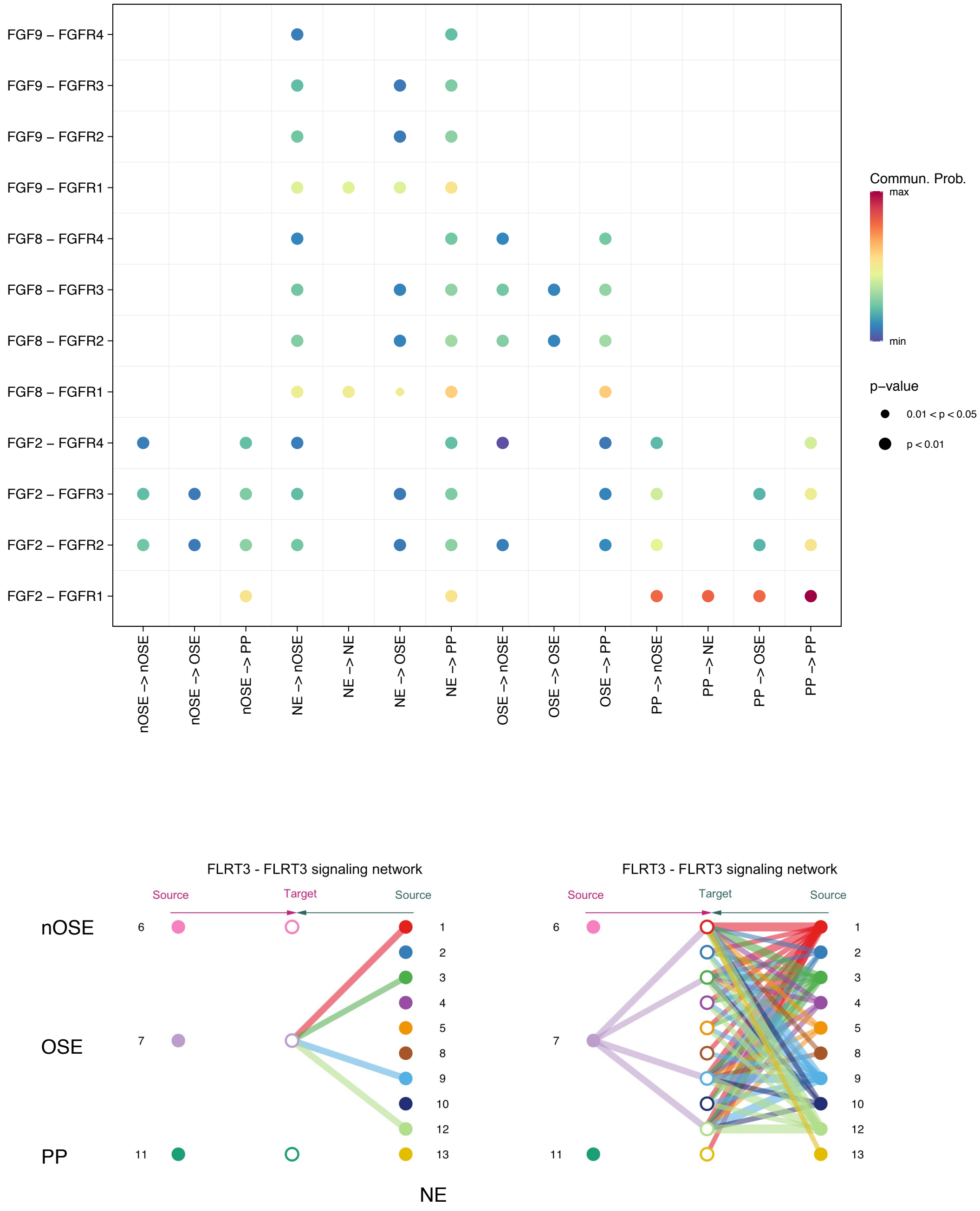

Figure S7: FGF and FLRT signalling in +1WK pre-SEAMs. (A) Bubble plot showing communication probabilities between specific FGF receptor-ligand pairs. (B) Hierarchy plot showing homogenic FLRT3-FLRT3 signalling. Target cells are shown in the centre of the hierarchy plots, and respective source cells at edges. Clusters 6, 7 and 11 represent nOSE, OSE and PP cells, and clusters 1-5, 8-12 and 13 are NE.

OSE, ocular surface ectoderm; nOSE, non-ocular surface ectoderm; NE, neuroectoderm; PP, pluripotent cells.

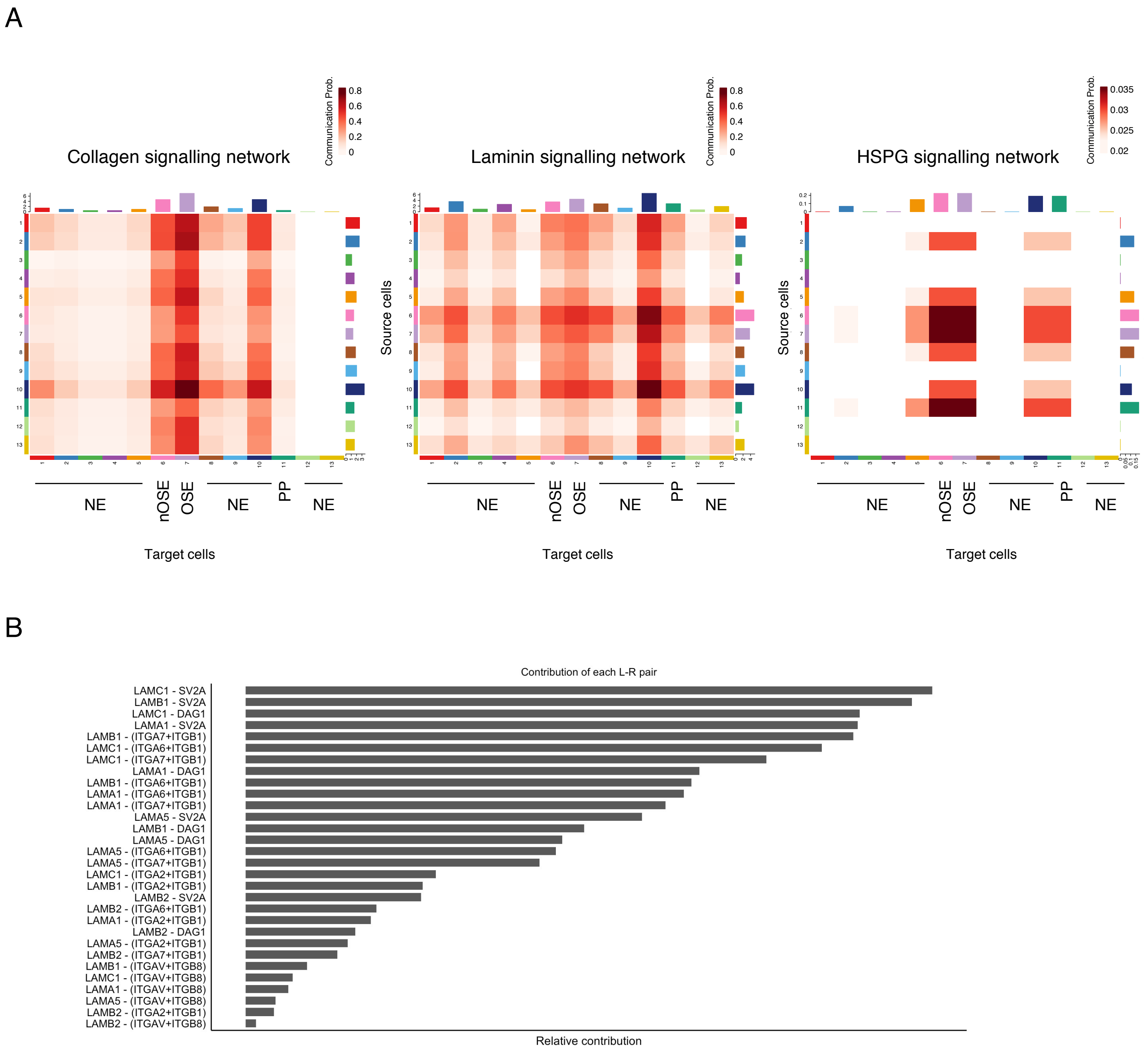

Figure S8: ECM signalling contribution in +1WK pre-SEAMs. (A) Heatmaps showing communication probabilities between specific collagen, laminin and HSPG receptor pairs for each cluster. (B) netAnalysis plot showing overall contribution of each laminin L-R pair In +1WK SEAMs, as shown by bar length. OSE, ocular surface ectoderm; nOSE, non-ocular surface ectoderm.

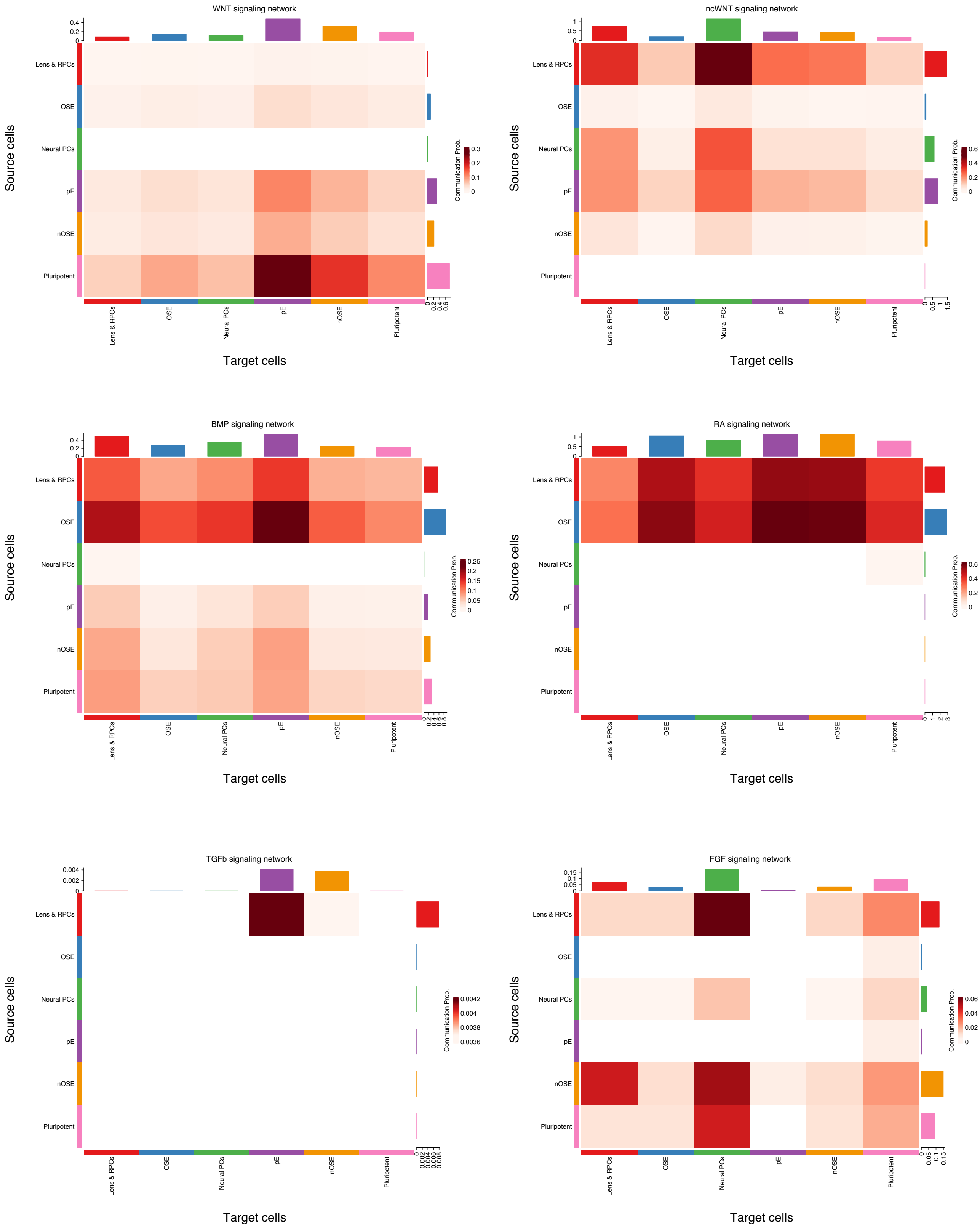

Figure S9: Signalling pathway contributions in +2WK SEAMs. Heatmaps showing communication probabilities in +2WK SEAMS. Cells were grouped according to developing cellular phenotypes. RPCs, retinal progenitor cells; OSE, ocular surface ectoderm; pE, proliferating SE; nOSE, non-ocular surface ectoderm.

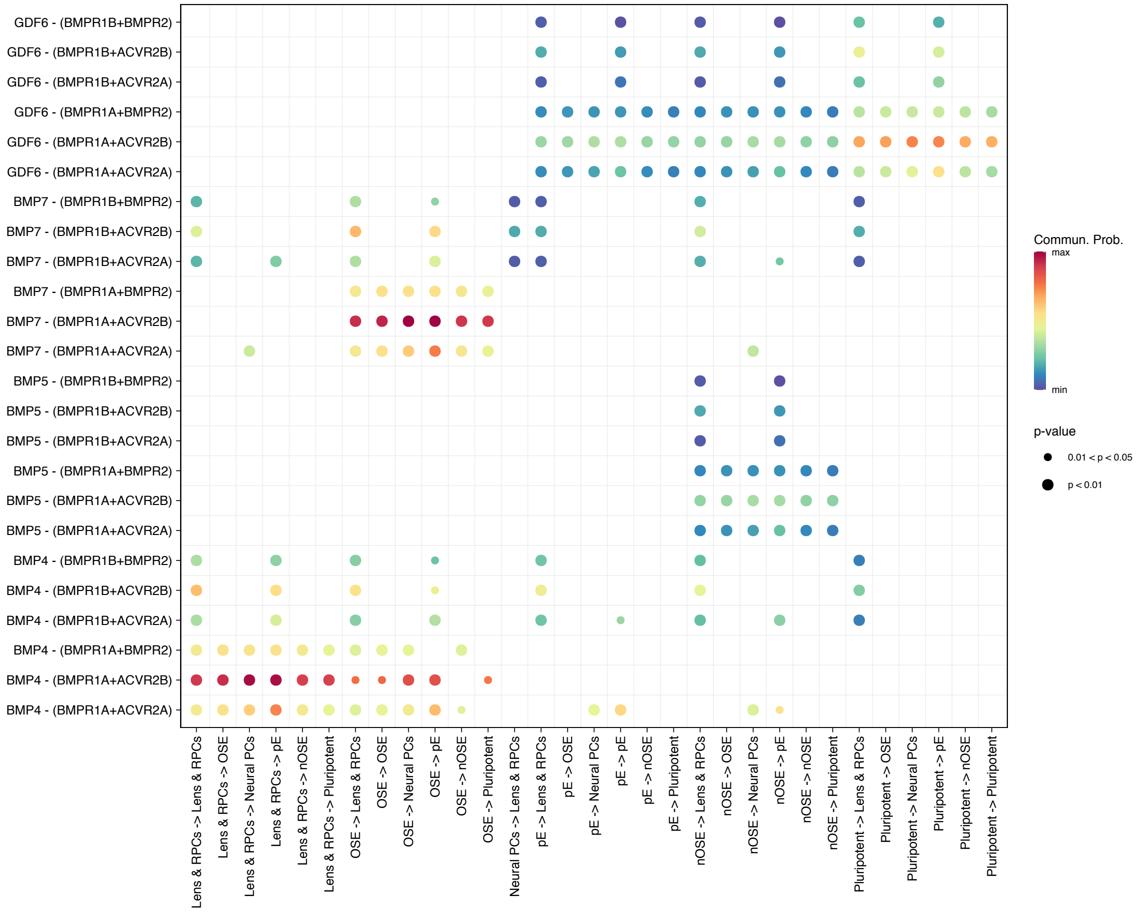

Figure S10: BMP receptor-ligand communication probabilities in +2WK SEAMs. Bubble plot showing communication probabilities between specific BMP receptor-ligand pairs. RPCs, retinal progenitor cells; OSE, ocular surface ectoderm; pE, proliferating SE; nOSE, non-ocular surface ectoderm.

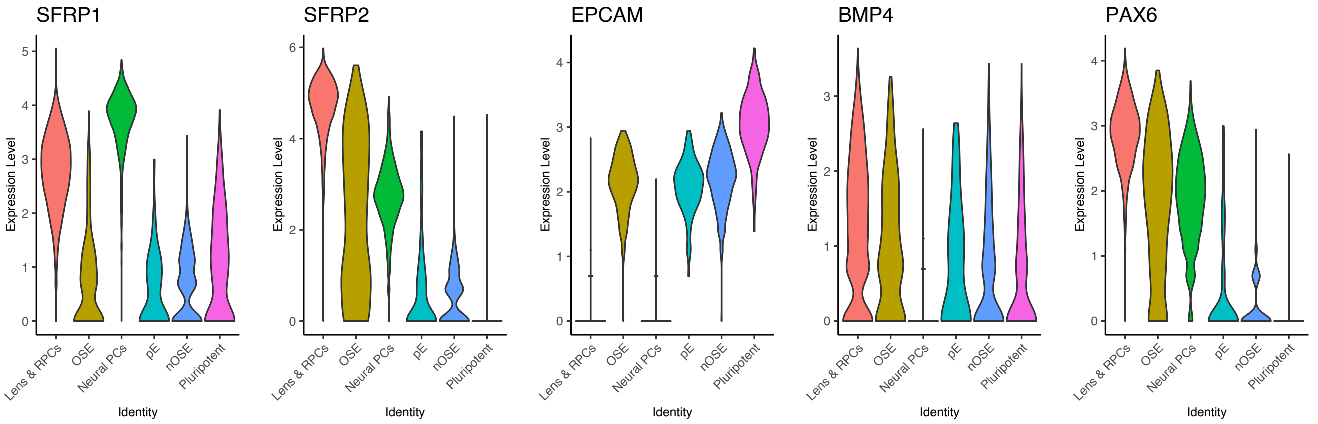

Figure S11: *SFRP1* and *SFRP2* in +2WK SEAMS. (a) Violin plots showing *SFRP1* and *SFRP2* expression in +2WK SEAMs. *EPCAM* and *BMP4* mark prospective non-neural ectoderm, and *BMP4* expression is also seen in lens & RPCs. High *PAX6* expression denotes ocular cells. RPCs, retinal progenitor cells; OSE, ocular surface ectoderm; pE, proliferating SE; nOSE, non-ocular surface ectoderm.

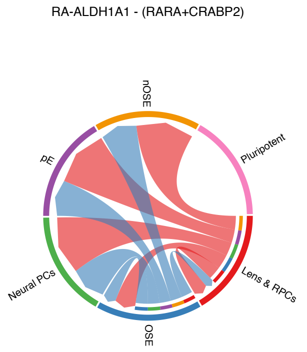

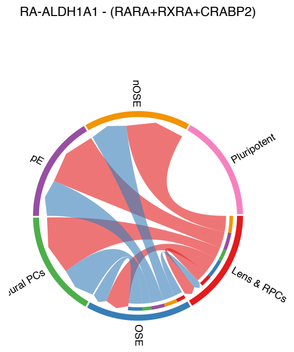

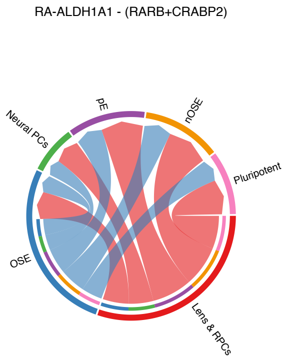

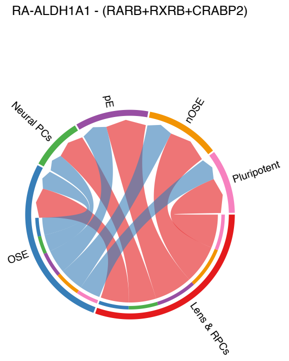

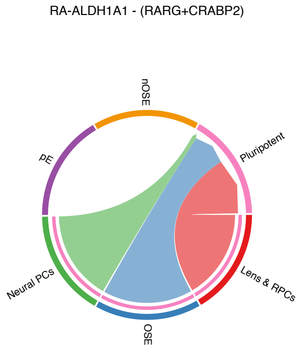

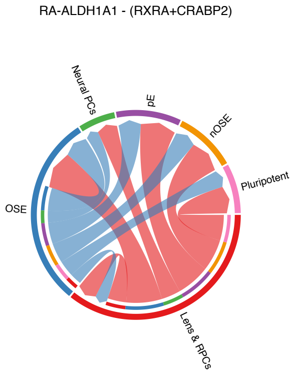

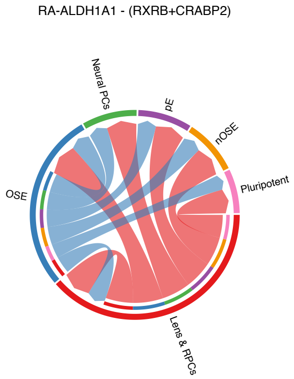

Figure S12: Individual RA ligand-receptor interactions in +2WK SEAMs. Chord plots showing contribution of specific RA L-R pairs, arranged according to cellular subtype. RPCs, retinal progenitor cells; OSE, ocular surface ectoderm; pE, proliferating SE; nOSE, non-ocular surface ectoderm.

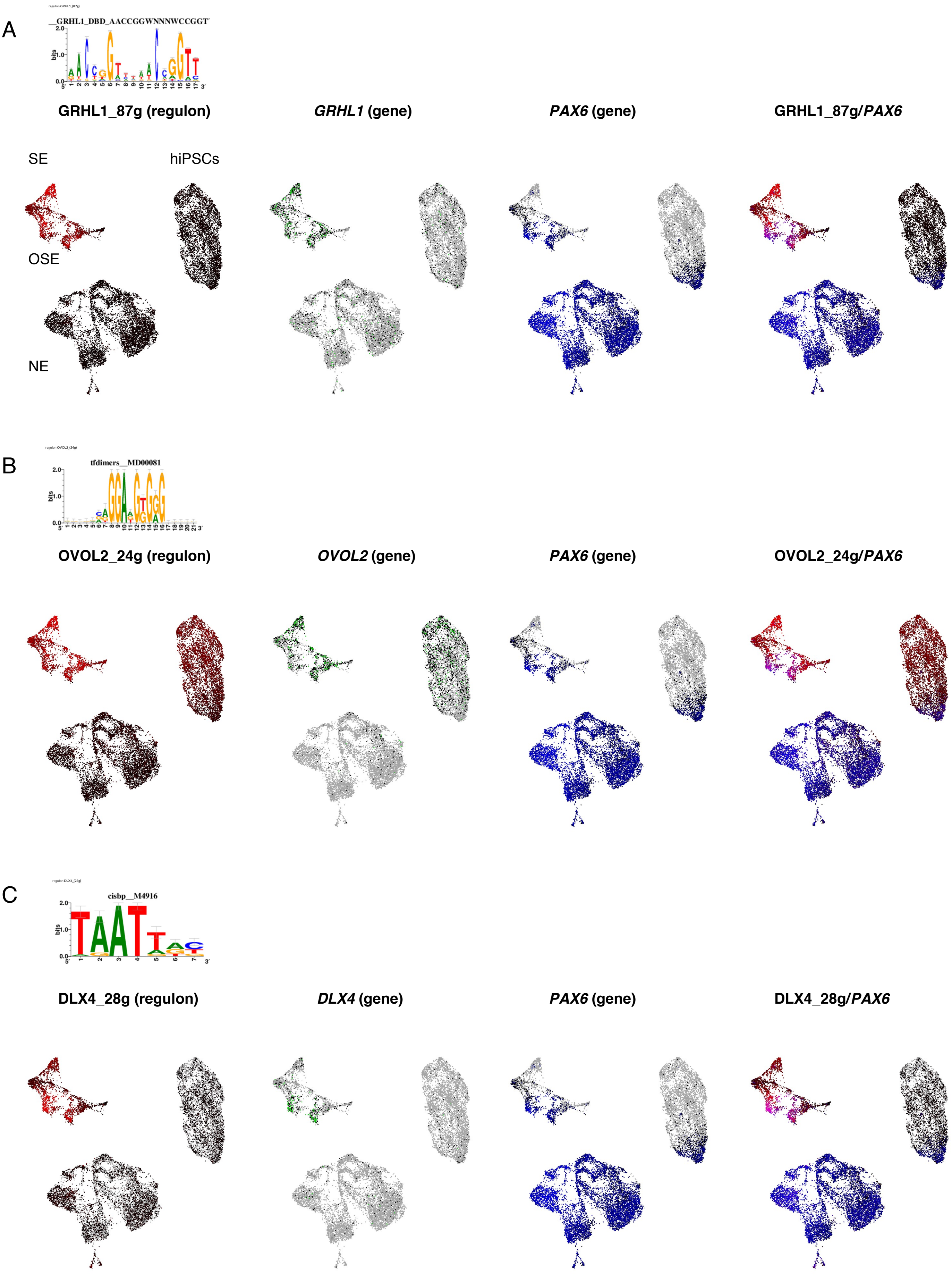

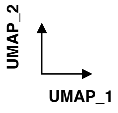

Figure S13. SCope visualisation of selected SCENIC-based transcription factor module activities. Regulon activity of (A)GRHL1_87g, (B) OVOL2_24g (C) DLX4_28g compared with expression domains. Binding motifs are indicated for each of the TFs.

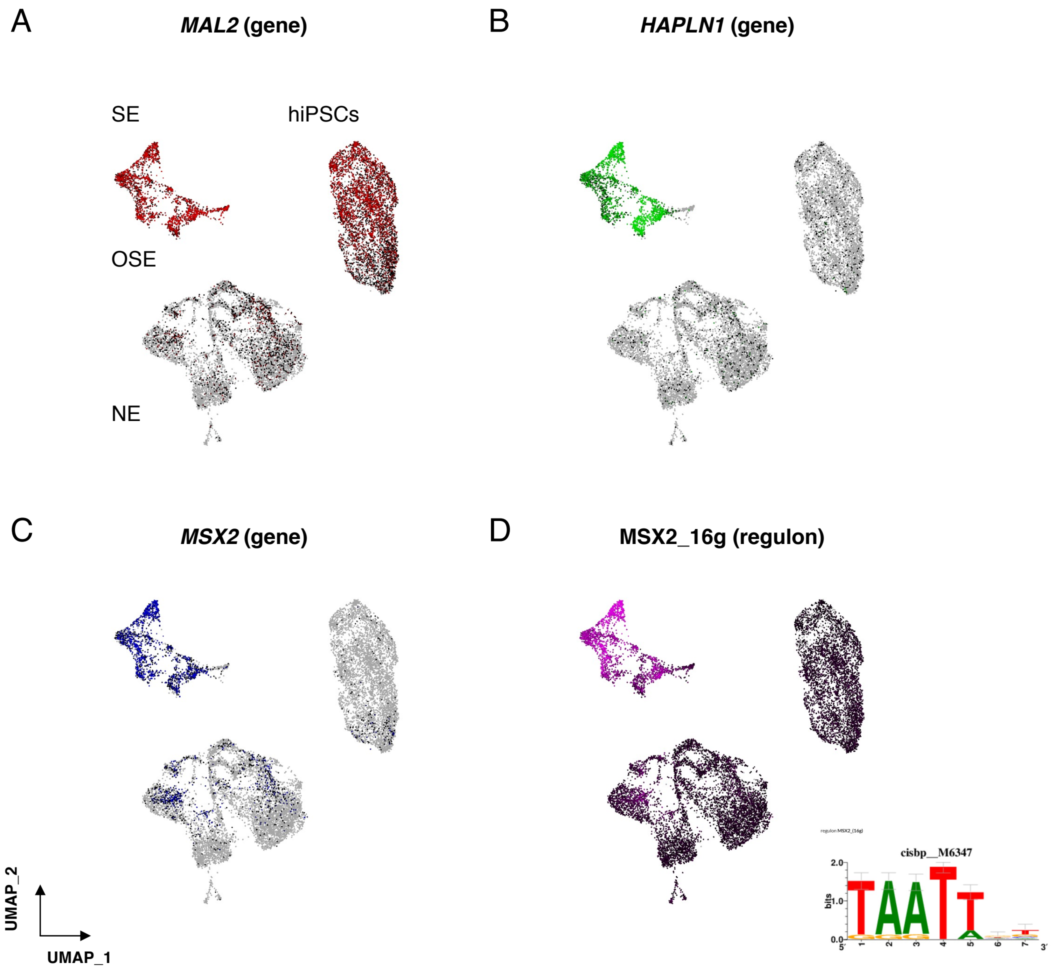

Figure S14. SCope visualisation of selected SCENIC-based TFs and target genes. Feature plots showing expression of (A) *MAL2*, (B) *HAPLN1* (C) *MSX2* plus (D) MSX2_16g regulon activity. Binding motifs are indicated for MSX2.
